# Secondary Bacterial Infections During Pulmonary Viral Disease: Phage Therapeutics as Alternatives to Antibiotics?

**DOI:** 10.3389/fmicb.2020.01434

**Published:** 2020-06-26

**Authors:** Prasanth Manohar, Belinda Loh, Sudarsanan Athira, Ramesh Nachimuthu, Xiaoting Hua, Susan C. Welburn, Sebastian Leptihn

**Affiliations:** ^1^Zhejiang University-University of Edinburgh Institute, Zhejiang University, Haining, China; ^2^The Second Affiliated Hospital, School of Medicine, Zhejiang University, Hangzhou, China; ^3^Antibiotic Resistance and Phage Therapy Laboratory, School of Biosciences and Technology, Vellore Institute of Technology, Vellore, India; ^4^Department of Infectious Diseases, Sir Run Run Shaw Hospital, School of Medicine, Zhejiang University, Hangzhou, China; ^5^Key Laboratory of Microbial Technology and Bioinformatics of Zhejiang Province, Hangzhou, China; ^6^Infection Medicine, Biomedical Sciences, Edinburgh Medical School, College of Medicine and Veterinary Medicine, The University of Edinburgh, Edinburgh, United Kingdom

**Keywords:** secondary bacterial infection, pulmonary viruses, SARS-CoV-2, COVID-19, phage therapy, phage endolysins

## Abstract

Secondary bacterial infections manifest during or after a viral infection(s) and can lead to negative outcomes and sometimes fatal clinical complications. Research and development of clinical interventions is largely focused on the primary pathogen, with research on any secondary infection(s) being neglected. Here we highlight the impact of secondary bacterial infections and in particular those caused by antibiotic-resistant strains, on disease outcomes. We describe possible non-antibiotic treatment options, when small molecule drugs have no effect on the bacterial pathogen and explore the potential of phage therapy and phage-derived therapeutic proteins and strategies in treating secondary bacterial infections, including their application in combination with chemical antibiotics.

## Introduction

The past two decades have seen the emergence of four severe viral outbreaks including the 2002 Severe Acute Respiratory Syndrome (SARS) Coronavirus (CoV) epidemic, the 2009 influenza A H1N1 pandemic, 2012 Middle East Respiratory Syndrome (MERS) outbreak, and most recently the COVID-19 pandemic. Emergence of novel lethal CoV strains in human populations are becoming more frequent and are of increasing global concern; SARS-CoV-2, a novel CoV that caused a first major outbreak in China in 2019 has now infected almost 8 million people globally and resulted in 434,000 deaths (as of June 15, 2020), a far greater disease burden than SARS and MERS (Guarner, 2020; Kannan et al., 2020). The spectrum of clinical presentations of COVID-19 is highly variable; infections range from being asymptomatic to severe viral pneumonia with respiratory failure, often leading to death (Li et al., 2020). During an epidemic, or pandemic, early development and roll-out of antiviral treatments that can reduce morbidity and mortality is critical. However, even with many potential repurposed and new anti-viral drug candidates able to inhibit replication or attachment of the virus, a major consequence of disease progression in patients at later stages of infection, are secondary bacterial infections. At least one in seven COVID-19 patients was found to be additionally infected with a secondary bacterial infection with 50% of the fatalities during the SARS-CoV-2 epidemic caused by untreated or untreatable secondary bacterial infections, in most cases in the lung (Zhou et al., 2020). While antibiotics do not have impact on the virus itself, almost all seriously ill patients are treated with antibiotics to attempt to prevent the occurrence of secondary bacterial infections. Any surge in antibiotic use during the COVID-19 pandemic will have a detrimental effect on antibiotic resistance rates for nosocomial bacterial pathogens, fueling global growth of antibiotic resistant bacterial pathogens (Reardon, 2020).

### Secondary Bacterial Infections

**FIGURE 1:**
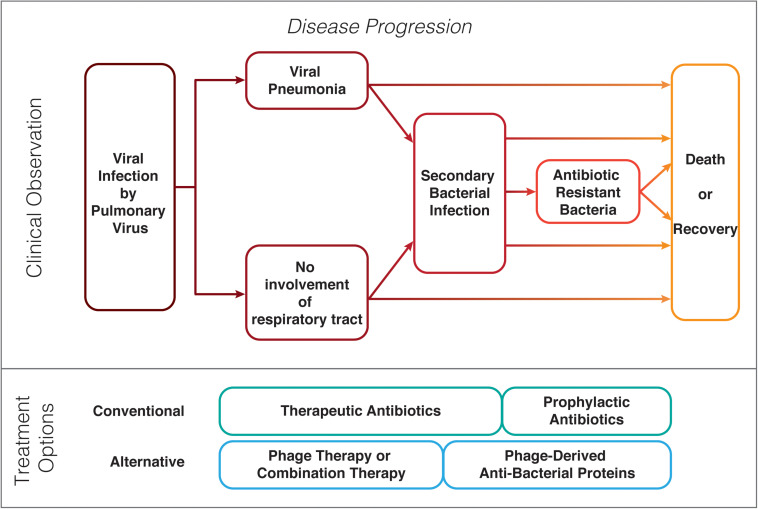
Possible clinical progression of patients with a pulmonary virus infection and prophylaxis or treatment options for bacterial secondary infections.

Secondary bacterial infections develop in patients during or after initial infection with an infective pathogen, often a virus (Morris et al., 2017; Wang et al., 2018) and are associated with high morbidity and mortality rates (Figure 1) (Mallia et al., 2012). Co-infections, secondary infections, or “superinfections” occur during viral epidemics; around 50 million deaths were ascribed to bacterial co-infections during the 1918–1919 Spanish Flu pandemic; although clinical records often do now record such infection complications (Kash and Taubenberger, 2015; MacIntyre et al., 2018). While secondary infections occur in succession to the primary infection, co-infections are caused by multiple pathogens of viral, bacterial, or fungal origin and occur simultaneously at the same time. There tends to be a strong focus on a single pathogen rather than a combination of pathogens, especially for the viral–bacterial infections most commonly observed in patients (Jamieson et al., 2010). *Staphylococcus aureus*, *Streptococcus pneumoniae*, *Neisseria meningitides*, *Haemophilus influenzae*, *Klebsiella pneumoniae*, and members of the genus *Proteus*, *Enterobacter*, and *Citrobacter* spp., are some of the most commonly isolated bacteria during secondary infections (Handel et al., 2009). Hospitals are a common source of the pathogens that cause secondary infections, these so-called nosocomial pathogen infections are acquired from an environment in which antibiotics are commonplace, and as such; many have acquired resistance to a broad range of antibiotics. Decades of misuse and, over-prescribing of antibiotics has resulted in selection of pathogens that show multi-drug resistance (MDR). MDR is a global health challenge as in many cases no (chemical) antibiotics exist to treat such infections, including secondary infections. Secondary bacterial infections are facilitated by the exposure to a pathogen together with an immune system that is inapt to appropriately react to both pathogen types, as a consequence of the primary viral infection. For such patients, the only option is to support their immune system and prevent progression of the infection that could lead to the death of the patient such as septic shock. Antibiotics therapies deployed as a “last resort” or the use of exceptionally high doses of antibiotics often have negative consequences. Many key human pathogens are showing resistance to antibiotics including Methicillin-resistant *S. aureus* (MRSA), multidrug-resistant *Streptococcus*, Vancomycin-resistant *Enterococci* (VRE), resistant *Mycobacterium*, Carbapenem-resistant *Enterobacteriaceae* (CRE), Colistin-resistant *Klebsiella*, Carbapenem-resistant *Pseudomonas aeruginosa*, and Carbapenem-resistant *Acinetobacter baumannii* (Kumar and Chordia, 2017). The problem is exacerbated by the discontinuation by big pharma of chemical antibiotics discovery programs in the search for chemical antibiotics (Loh and Leptihn, 2020). Other options to treat MDR infections would be beneficial.

### Molecular Basis of Manifestation of Secondary Bacterial Infections

There are several hypotheses why secondary infections quickly establish in patients with a pulmonary viral infection, including immunological host changes, mechanical damage and diffusion, and removal of mucus within the lungs. Much of how secondary infections manifest themselves and what role the immune response to a virus influences the defense against a prokaryotic pathogen, is poorly understood. The prevalence of secondary bacterial infections during a primary virus disease is due to the altered immune response to one pathogen (here: the virus) that often changes the response of the system to the other infectious agent (here: the bacterium), resulting in increased morbidity (Hendaus et al., 2015). Severe viral infections initiate changes in the immune response of the host that persist for a prolonged period of time. These immune alterations termed “trained immunity,” “innate imprinting,” or “immune paralysis,” change the inflammatory response of immune cells (Williams et al., 2004; Bordon, 2014; Cheng et al., 2014; Roquilly et al., 2017; Morgan et al., 2018). In addition, viral infections of the respiratory system can lead to immune responses that alter the microbiome of the host. This change in the microbiome has been suggested to possibly modulate immune cell priming against secondary bacterial challenge (Hanada et al., 2018).

In most cases, epithelial cells are damaged during the primary viral infection, impeding mucociliary clearance that leads to an accumulation of mucus (Perry et al., 2005). Bacteria are able to diffuse into the mucus but thickened dense mucus impedes penetration of host immune cells. Secondary bacterial infections are also facilitated by the immune response of the host to a viral attack. One of the more important factors seems to be the immunosuppression of the host innate immune response initiated by the viral infection which facilitates opportunistic bacteria, like *Streptococcus* to infect the host (Kim et al., 2011). Cells of a host suffering from a viral infection are more susceptible to bacterial attachment and colonization (Pittet et al., 2010; Nyangacha et al., 2017). Many viruses, including influenza virus, rhinovirus, and respiratory syncytial virus (RSV), have detrimental effects on the mucosal layer facilitating bacterial adherence of, e.g., *S. pneumoniae*, *P. aeruginosa*, and *H. influenzae*, as well as biofilm formation on the linings of the lungs (Morris, 2007). For some pulmonary viruses such as influenza, Toll-like receptor (TLR) pathways are altered, possibly from sustained de-sensitization, which results in an increase of attachment of bacteria to epithelial cells (Kurt-Jones et al., 2000; Haynes et al., 2001). TLR and RIG-I-like receptor activation results in the production of Type I Interferon, changing the inflammatory response to TLR ligands, e.g., bacterial lipopolysaccharide (Doughty et al., 2001; Nansen and Thomsen, 2001). Polymicrobial, viral–bacterial co-infections can develop as a result of an altered immune response combined with accessible routes of entry for the bacterial pathogen(s).

For patients with viral–bacterial infections, the availability of therapeutic options for both infectious agents is crucial. Antivirals are deployed to combat the virus, which have no effect on the bacterial pathogens (McCullers, 2011), and bacterial infections are treated with antibiotics, or such small-molecule compounds to try and prevent secondary infections. Broad-spectrum antibiotic may result in undesirable inflammatory responses in the afflicted person (Brook, 1995, 2002; Mahar et al., 2014). To prevent complications caused by secondary bacterial infections and to eliminate bacterial pathogens, alternative antibacterial therapies are needed.

### Coronavirus, Pneumonia, Antibiotics, and Antibiotic Resistance

Some of the most significant outbreaks, epidemics, and global pandemics, with high morbidities and mortality are from viral respiratory infections caused by influenza virus or CoV species; for which treatment is compromised by secondary bacterial infections (Yang et al., 2020; Zhou et al., 2020). While a viral infection alone can be detrimental to a patient, the pathogen that exasperates disease progression is most commonly of bacterial origin (Yang et al., 2020). During the 2009 H1N1 influenza pandemic, between 29 and 55% of fatal cases were caused by secondary bacterial infections (Center for Disease Control and Prevention, 2012). During the ongoing COVID-19 pandemic, around 15% of hospital cases have been associated with secondary bacterial pathogens, and 50% of patients died (Zhou et al., 2020). Clinical cases of COVID-19 have developed high rates of up to 50% of secondary bacterial infections leading to secondary bacterial pneumonia. Half of COVID-19 fatalities experienced some form of secondary infection (pulmonary or other) that may have contributed to their death. During severe COVID-19 disease with pneumonia, the air sacs of the lungs fill with pus and fluids, nutritious substrates for pathogens including *P. aeruginosa* and *S. aureus*. Tissues are breached by the cytolytic activity of the virus and bacteria invade deeper into the tissue, continuing to secrete toxins that further destroying surrounding cells. The frequency (30–40%) of complications due to bacterial involvement was significantly higher in fatal COVID-19 patients than for COVID-19 survivors (Zhou et al., 2020). In fatal COVID-19 cases, death is most frequently a result of respiratory failure from severe pneumonia, caused either by SARS-CoV-2 itself or as a result of a secondary bacterial infection (Tetro, 2020).

Patients suffering of a pulmonary virus infection, including COVID-19, are often administered prophylactic antibiotics, including azithromycin, moxifloxacin, ceftriaxone, vancomycin, or cefepime, to reduce the risk of secondary infections; often in addition to another antibiotic that is deployed once the infection is identified (Holshue et al., 2020; Wang et al., 2020). As the numbers of antibiotic-resistant bacterial strains continue to grow, there is increased the risk of superinfection in severely ill patients, especially in intensive care units (ICUs). During the COVID-19 pandemic, many clinical case studies of COVID-19 patients have been reported, but mainly these focus on the viral infection itself. There are few reports detailing secondary bacterial infections and even less describing AMR. Until a vaccine is deployed globally, bacterial secondary infections will continue to be important in COVID-19 clinical care. New antibiotics or alternative treatments targeted against secondary bacterial infections need to be developed for COVID-19 and subsequent pandemics.

### Antibacterial Therapy in Patients With Non-bacterial Primary Infections

To reduce the risk of superinfections, cases of pneumonia caused by respiratory viruses including SARS-CoV-2 are often prophylactically treated with antibiotics that target a broad-spectrum of bacteria. The alternative, i.e., no prophylactic treatment, often results in bacterial infections, in principle, demonstrating the effectiveness of this strategy. Prophylactic use of antibiotics, however, contributes to the AMR crisis and is ineffective when patients acquire antibiotic-resistant strains—nosocomial hospital-acquired infections are commonly observed for ICU patients infected with a respiratory virus (Zhou et al., 2020). Nosocomial infections are becoming more common due to the rise of resistant bacterial pathogens. Alternative antibacterial therapeutic strategies are needed; such as phage therapy or phage-derived therapeutic proteins.

### Phage Therapy and Phage-Based Strategies During Viral Epidemics

**FIGURE 2:**
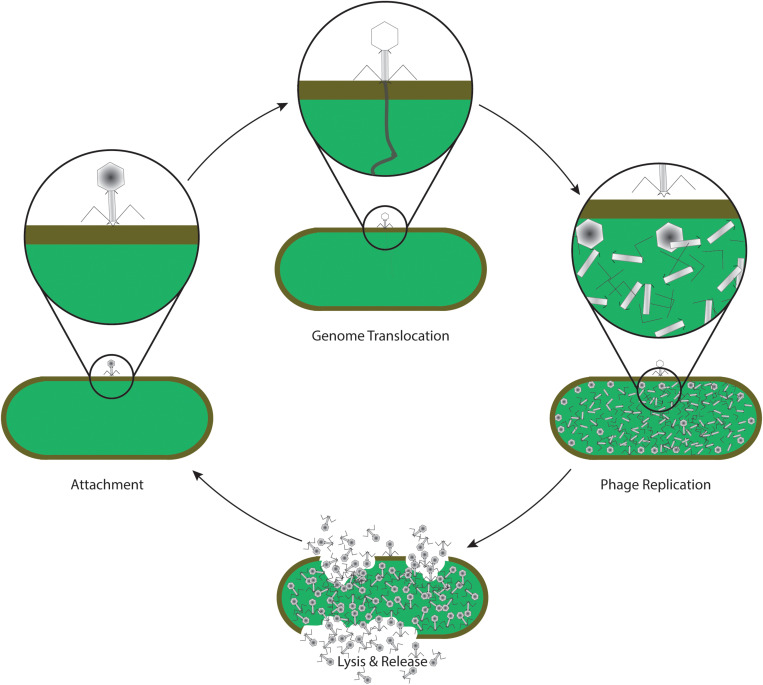
Life cycle of a lytic phage, commonly used in phage therapy. After specific recognition and attachment to the host cell (attachment), phage proteins insert into the envelope of the cell, forming a channel through which the viral genome is translocated into the cytoplasm of the bacterium (genome translocation). The host machinery replicates the genomic information, and synthesizes phage proteins which self-assemble to form the virus structure (phage replication). Later in the life cycle, specific phage proteins such as holins and endolysins are synthesized. Enzymatic activity of endolysins leads to a disintegration of the host envelope, resulting in host cell lysis and release of phage progeny (lysis and release). The new phages are now able to attach and infect new host cells again, continuing the life cycle.

**FIGURE 3:**
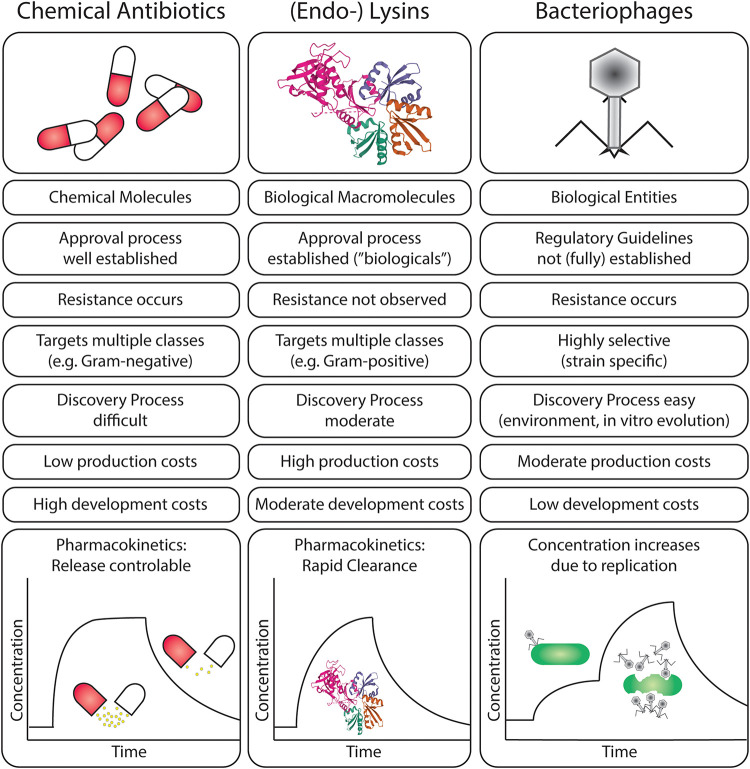
Comparison of scientific, clinical, and pharmaceutical characteristics of chemical antibiotics, endolysins, and bacteriophages in the treatment of bacterial infections.

An alternative approach to treating secondary bacterial infections, especially AMR pathogens, is the application of phage therapy; using microbial viruses to kill their host, ideally clearing the bacterial infection (Altamirano and Barr, 2019). Bacteriophages are naturally occurring viruses that use bacteria as hosts after employing the host machinery for propagation, including genome replication, protein synthesis, and phage assembly (Figure 2). Lysogenic bacteriophages undergo a “dormant” state as non-replicative, genome-integrated phages (Monteiro et al., 2019; Hampton et al., 2020). Some filamentous phages allow the host to continue to grow and divide while the phages are being produced (Loh et al., 2017, 2019; Kuhn and Leptihn, 2019). Phage therapy was discovered before antibiotics but the opportunities from the “dawn of the antibiotic era” delayed pursuit of commercial therapeutic phage strategies (Pirisi, 2000; Housby and Mann, 2009; Manohar et al., 2019c). With increasing AMR, and depletion of antibiotic resources, phage therapy has once more piqued the interest of the scientific community and pharmaceutical industry (Seguin et al., 2006; Nyangacha et al., 2017; Manohar et al., 2019c; Loh and Leptihn, 2020). Phages and phage-derived therapeutic proteins have advantages and disadvantages as compared with antibiotic therapies (Figure 3).

A major advantage of phages is that they exhibit bacterial host specificity (Seguin et al., 2006) and selectively target pathogenic bacteria without adverse effects on the normal microflora (Brüssow, 2005). Phages may be genus- or species-specific, and even strain-specific, infecting and killing their target bacterium. As most secondary bacterial infections are caused by one bacterial genus, species or strain, phage therapy offers a promising treatment option. Clinical studies using phages have demonstrated success at eliminating resistant bacterial strains (Capparelli et al., 2007; Kutter et al., 2010; Chan et al., 2013). Intact phage particles do not adversely interact with human cells and are not able to cause infections in humans (Barr, 2019; Van Belleghem et al., 2019). The self-replicating nature of phages enables them to function as “active and self-replicating” drugs once administered to the patient so dosage is less important to reach therapeutic levels; however—in practice—a large quantity of phages are being deployed (Barr, 2019). Phages show low toxicity; being mainly composed of protein and DNA and eventually undergo degradation inside a patient’s body. Degradation of phage particles does not result in the production of toxic molecules, unlike antibiotics that could apply strain on the liver and might result in organ failure. Since most secondary bacterial infections occur in immunocompromised or immunodeficient patients, it is necessary to study the efficacy of phage therapy under such conditions. Previous studies on cancer patients and renal allograft patients showed that phage therapy can be effective in treating secondary bacterial infections and co-infections (Weber-Dabrowska et al., 2001; Miȩdzybrodzki et al., 2012). Phages often do not cause an immune response, in contrast to human-pathogenic viruses (Manohar et al., 2019a). The levels to which administered phages can induce immune responses have been well studied and no adverse side effects have been observed (Borysowski and Górski, 2008; Ahmadi et al., 2016; Jault et al., 2019; Voelker, 2019). While there is a lack of evidence showing the efficacy of phage therapy during viral infections, bacteriophages have been shown to be effective prophylactic agents (Ahmadi et al., 2016).

### Synergistic Effects of Phages and Antibiotics

A number of factors can result in phage resistance in bacteria, such as the alteration in phage receptors on the bacterial cell surface; degradation of phage DNA by restriction endonuclease produced by the host bacteria; emergence of mechanisms inhibiting the penetration of phage DNA into the bacterial host, among others (Jo et al., 2016b; Valério et al., 2017). Under certain conditions, such as low phage to bacterium ratios, or when using a single phage to treat an infection, phage-resistant bacteria can emerge. This selection of resistant mutants is a shortcoming of phage therapy. Clinical applications often make use of multiple phages, making the occurrence of multiple resistance mechanisms unlikely (Summers, 2001; Lin et al., 2017).

Another solution to overcome such limitations is to conduct combination therapy. The combined use of phages and antibiotics is expected to provide stronger suppression of bacterial growth and is expected to help reduce bacterial resistance against phages and antibiotics (different targets are used by the two agents). Recent *in vitro* and *in vivo* studies have shown improved efficacy in controlling the growth of bacterial pathogens such as *Pseudomonas fluorescens*, *Pseudomonas aeruginosa*, *Escherichia coli*, and *S. aureus* using combinations of phages and antibiotics (Torres-Barceló et al., 2016; Chaudhry et al., 2017). Phage-antibiotic therapy is reported to have higher success rates in preventing the emergence of bacterial resistance as bacteria that become non-susceptible to one agent can still be killed by the other and *vice versa*. Phage-antibiotic therapy can prevent the emergence of double resistant bacterial mutants, as a bacterium is unlikely to acquire phage and antibiotic resistance simultaneously. For the success of combination therapies, it is essential to carefully choose the dosage as well as the point of administration of each antibacterial agent. Administration of antibiotics prior to the phage resulted in the decreased evolution of phage resistance due to antibiotic stress (Torres-Barceló, 2018; Tagliaferri et al., 2019). Phage-antibiotic combinations that have different bacterial targets can assist in enhanced bacterial inactivation.

The use of sub-lethal concentrations (concentration of antibiotics lower than minimal inhibitory concentrations) of antibiotics can enhance phage productivity mediating phage induced bacterial decline (Tagliaferri et al., 2019), known as phage-antibiotic synergy. One factor proposed responsible for phage-antibiotic synergy is bacterial elongation or filamentation induced by sub-lethal concentrations of antibiotics (Kim et al., 2018). While different antibiotics exhibit different inhibitory mechanisms, they often result in the blocking of bacterial cell division causing elongation of the bacterial cell, as a large bacterial surface area is available for phage attachment, bacterial cells are vulnerable toward phage infection, increasing the efficacy of phage lytic activity (Comeau et al., 2007; Kim et al., 2018). Antibiotic-induced morphological changes in the host bacterium can permit faster phage assembly due to altered or abundant precursors required for phage maturation, which ultimately can result in an acceleration of cell lysis (Nouraldin et al., 2016). Beta-lactam antibiotics and quinolones can stimulate virulent phage production, together with the changes induced in bacterial membrane proteins by antibiotics and may enhance the lytic activity of phages and increase phage burst size observed in phage-antibiotic studies (Jo et al., 2016a). A further advantage of using sub-inhibitory concentrations of antibiotics is avoidance of side effects from administration of otherwise necessary higher doses of the antibiotic (Knezevic et al., 2013).

Infections caused by biofilm-forming bacteria, especially nosocomial infections, are difficult to treat using antibiotics (Pires et al., 2017). The emergence of ventilator-associated secondary pneumonia is often due to biofilm forming bacteria such as *Pseudomonas* and *Staphylococcus* (Zhou et al., 2020). Phage-antibiotic therapy has shown promise for biofilms and in embedded bacteria; phages can enzymatically penetrate the microenvironment created by biofilm-forming cells to infect and kill target bacteria (Pires et al., 2017). Phages, when administered in combination with an antibiotic, can increase the susceptibility of bacteria and reduce plasmid-borne bacterial resistance by targeting plasmid bearing bacteria in the biofilm (Łusiak-Szelachowska et al., 2020). Combined phage and antibiotic therapy with a sequential approach of administering the antibiotic after phage treatment of the biofilm has shown success; initial treatment of the biofilm with phages caused biofilm disintegration, while later administration of the antibiotic prevented biofilm regrowth as well as the emergence of phage-resistant mutants. Phages can enable antibiotics to penetrate biofilm layers by degrading the biofilm matrix; phages penetrate into deeper layers of the biofilm, replicating in the lower layers and facilitating the destruction of the biofilm (Łusiak-Szelachowska et al., 2020). Antibiotics administered following biofilm treatment by phages enhance bacterial reduction Akturk et al., 2019.

### Phage Endolysins as Antibacterial Agents

**FIGURE 4:**
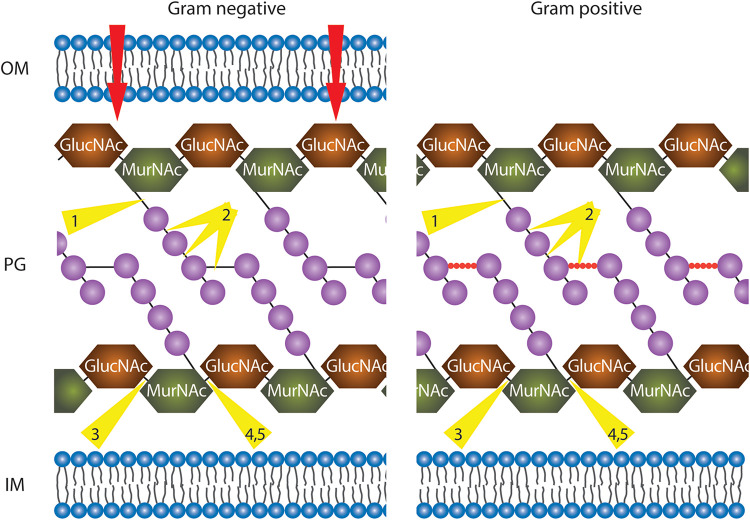
Illustration of phage endolysins classified by their enzymatic activity. In order to reach the peptidoglycan (PG) layer, in the case of Gram-negative bacteria, the outer membrane (OM) has to be traversed (red arrows, details in text). The PG layer is represented with alternate repeating sugar units, *N*-acetylglucosamine (GlucNAc), and *N*-acetylmuramic acid (MurNAc). In Gram-negative bacteria, tetrapeptide chains (purple spheres) extend from each MurNAc of the long sugar chains and are directly cross-linked by short interpeptide bridges (black solid line) via the third residue to D-Ala of another peptide chain. In Gram-positive bacteria, the tetrapeptide chains (purples spheres) are linked via pentapeptide bridges (red spheres). All residues are purple spheres. IM, inner membrane. Depending on the organism, residues at position 3 are either L603 Lysine (mainly Gram-positive bacteria) or meso-diaminopimelic acid (Gram-negative bacteria and Gram-positive bacilli). Endolysin cleavage sites are indicated by yellow triangles: 1, *N*-acetylmuramoyl-L-alanine amidases; 2, endopeptidases; 3, endo-*N*-acetyl606 glucosaminidases; 4, *N*-acetyl muramidases; 5, lytic transglycosylases.

Phage proteins can be extracted and also engineered for deployment as an alternative to whole phage therapeutic applications. Endolysins are phage-encoded enzymes produced at the end of the bacteriophage lytic lifecycle that facilitate release of phage progeny into the environment, degrading the peptidoglycan layer of the host bacterial cell wall (Imanishi et al., 2019). The ability of endolysins to digest the bacterial cell wall when applied exogenously offers the opportunity for their deployment as potential antibacterial agents, to target and kill pathogenic bacteria, potentially without harming the normal microflora due to specificity of enzyme activity (Haddad Kashani et al., 2017). The chances of bacteria developing resistance toward endolysins are low since endolysins target cell wall molecules essential for bacterial viability (Łusiak-Szelachowska et al., 2020). Phage-derived endolysins are promising candidates for treating secondary bacterial infections or multi-drug resistant infections (Imanishi et al., 2019; Kim et al., 2020). There are five main classes of endolysins based on their enzymatic specificity (Figure 4) (Schmelcher et al., 2012). Several applications for endolysins have been reported, including use in biocontrol agents against bacterial pathogens in food and agricultural industries (Matamp and Bhat, 2019) and as strong antibacterial agents for human pathogens in animal models (Guo et al., 2017; Haddad Kashani et al., 2017; de Wit et al., 2019). Most naturally occurring endolysins have limited effect on Gram-negative bacteria as the outer membrane (OM) protects the peptidoglycan layer from being attacked by the enzyme. However, efficient endolysin-mediated inactivation of Gram-negative pathogens can be achieved by either chemical/physical permeabilization of the OM (such as pressure or chelation of divalent ions), or by creating endolysin-peptide-fusions that have the ability to permeabilize the OM (Briers and Lavigne, 2015).

The application of bacteriophages in therapy has been successful in treating: (i) MDR *A. baumannii* infections (Schooley et al., 2017); (ii) against Mycobacterial infections (Dedrick et al., 2019); (iii) burn-wound infections caused by *P. aeruginosa* (Jault et al., 2019); (iv) infections caused by *S. aureus* (Fabijan et al., 2020); (v) Enterococcal infections (Khalifa et al., 2016); and (vi) against MDR *E. coli* infections (Sarker et al., 2016; Kakasis and Panitsa, 2019). Preparations of phage cocktails, phage banks, phage powders, and phage-derived endolysins as part of phage therapeutic approaches are possible and bring strategies closer to clinical applications (Manohar et al., 2019b,c).

### Hurdles for Deployment of Phages

With the looming crisis of increasing numbers of MDR bacterial infections, alternatives to chemical antibiotics are urgently needed. To prevent secondary bacterial infections, prophylactic use of phages could be applied similar to small-molecule broadband antibiotics. This would require a “phage cocktail” that targets a broad range of pathogenic species likely to cause bacterial pneumonia. Since phages are highly specific, a phage cocktail would need to contain a variety of phages. Regulatory guidelines for the medical application of phages are not (fully) established in most countries; each component of a therapeutic requires to be approved, making deployment of phages as prophylactics in patients infected with pulmonary viruses, complex. Phage-derived therapeutic proteins such as endolysins would be advantageous as they would have a lower specificity toward bacteria and be able to inactivate a broader range of bacterial pathogens.

Phages can also be used as therapeutic agents for an established bacterial infection. Due to the high specificity of phages, phage therapy can be considered a personalized medicine (Loh and Leptihn, 2020). However, a phage to which the causative pathogen is susceptible would need to be rapidly identified. To treat multi-drug resistant pathogens, and to be prepared for the next pandemic where such infections, especially nosocomial ones, have a major impact on patient survival, phage libraries, and rapid on-site (hospital) screening platforms should be made available for use.

While phage therapy is unlikely to replace chemical antibiotics in clinical practice, it offers a potential solution to be used in combination with antibiotics or alone. Limitations of phage therapy in treating secondary bacterial infections include: the preparation of therapeutic cGMP (Good Manufacturing Practice) phages; a lack of proper clinical trials or guidelines to treat patients in different clinical conditions; a lack of regulatory guidelines to prepare and administer phages; patient acceptance, and possibly, adverse immune responses leading to primary treatment failure.

## Conclusion

Secondary bacterial infections play a critical role in the morbidity and mortality rates of patients initially falling ill with pulmonary viral diseases. Evidence from the current SARS-CoV-2 pandemic shows that the antibiotic-resistant bacterial infections are a significant threat to hospitalized COVID-19 patients. Nosocomial infections including ventilator-associated infections are often unavoidable and especially so during a pandemic, and the use of broad-spectrum antibiotics is often a routine preventative measure. Phage therapy is one of the most promising options for treating secondary bacterial infections. Phage therapy either as a stand-alone treatment or in combination with antibiotics may offer a valuable alternative for treating secondary bacterial infections. Clinical studies should evaluate the efficacy of phage therapy in virus infected patients.

The potential for application of phage products, such as endolysins, should be investigated. Pulmonary CoVs will likely be a clinical challenge for many years to come. Viral pandemics from CoVs and emerging pathogens are inevitable in our globalized world with interconnected societies, travel, and commerce. We require to be well prepared for long-term management of COVID-19 and for the next pandemic, exploring and establishing new avenues to treat bacterial pathogens commonly observed in secondary infections. To avert an emerging healthcare crisis due from COVID-1 and antibiotic-resistance of secondary infections; medical interventions employing phage products or phage therapy itself might be our most promising options.

## Author Contributions

PM contributed to the initial idea. PM, BL, SW, and SL contributed to the concept and writing. BL and SL prepared the figures. SA, RN, and XH contributed to the review and comments.

## Conflict of Interest

The authors declare that the research was conducted in the absence of any commercial or financial relationships that could be construed as a potential conflict of interest.
